# Are Endomyocardial Ventricular Biopsies Useful for Assessing Myocardial Fibrosis?

**DOI:** 10.3390/jcm13113275

**Published:** 2024-05-31

**Authors:** Igor Makarov, Daria Voronkina, Alexander Gurshchenkov, Anton Ryzhkov, Anna Starshinova, Dmitry Kudlay, Lubov Mitrofanova

**Affiliations:** 1 Almazov National Medical Research Centre, St. Petersburg 197341, Russia; drvrnkn2003@gmail.com (D.V.); gurshchenkov_av@almazovcentre.ru (A.G.); ryzhkov_av@almazovcentre.ru (A.R.); mitrofanova_lb@almazovcentre.ru (L.M.); 2 Department of Pharmacognosy and Industrial Pharmacy, Faculty of Fundamental Medicine, Lomonosov Moscow State University, Moscow 119991, Russia; d624254@gmail.com; 3 Institute of Immunology FMBA of Russia, Moscow 115478, Russia

**Keywords:** collagen volume fraction, myocardial fibrosis area, endomyocardial biopsy, chronic myocarditis, hypertrophic cardiomyopathy, heart failure, COVID-19

## Abstract

Myocardial fibrosis is an important factor in the progression of cardiovascular diseases. However, there is still no universal lifetime method of myocardial fibrosis assessment that has a high prognostic significance. The aim of the study was to determine the significance of ventricular endomyocardial biopsies for the assessment of myocardial fibrosis and to identify the severity of myocardial fibrosis in different cardiovascular diseases. **Material and Methods:** Endomyocardial biopsies (EMBs) of 20 patients with chronic lymphocytic myocarditis (CM), endomyocardial fragments obtained during septal reduction of 21 patients with hypertrophic cardiomyopathy (HCM), and 36 patients with a long history of hypertensive and ischemic heart disease (HHD + IHD) were included in the study. The control group was formed from EMBs taken on 12–14 days after heart transplantation (n = 28). Also, for one patient without clinical and morphological data for cardiovascular pathology, postmortem myocardial fragments were taken from typical EMB and septal reduction sites. The relative area of fibrosis was calculated as the ratio of the total area of collagen fibers to the area of the whole biopsy. Endocardium and subendocardial fibrosis were not included in the total biopsy area. **Results:** The relative fibrosis area in the EMBs in the CM patient group was 5.6 [3.3; 12.6]%, 11.1 [6.6; 15.9]% in the HHD + IHD patient group, 13.4 [8.8; 16.7]% in the HCM patient group, and 2.7 [1.5; 4.6]% in the control group. When comparing the fibrosis area of the CM patients in repeat EMBs, it was found that the fibrosis area in the first EMBs was 7.6 [4.8; 12.0]%, and in repeat EMBs, it was 5.3 [3.2; 7.6]%. No statistically significant differences were found between the primary and repeat EMBs (*p* = 0.15). In ROC analysis, the area of fibrosis in the myocardium of 1.1% (or lower than one) was found to be highly specific for the control group of patients compared to the study patients. **Conclusions:** EMB in the assessment of myocardial fibrosis has a questionable role because of the heterogeneity of fibrotic changes in the myocardium.

## 1. Introduction

Myocardial fibrosis is the most common pathologic process in cardiac tissue. It represents the final phase of tissue response to myocardial damage of any origin, whether due to pressure overload in the context of hypertension (HHD) or as a result of direct and/or mediated tissue damage in chronic myocarditis [1].

Oxidative stress, the final common pathway of cellular damage observed in various cardiovascular diseases, is of great importance for the initiation of fibrosis. The accumulation of oxidation products and the inability to adapt to the stress caused by reactive oxygen species lead to the activation of the immune system. This leads to the development of proinflammatory and profibrotic states causing functional and structural changes in myocardial cells and interstitium [2].

Structural changes in the myocardium and vascular wall, of which fibrosis is a key component, are the basis for the progression of most cardiovascular diseases (CVDs), including heart failure syndrome (HF). A study conducted by Wong, T. C. et al. using magnetic resonance imaging (MRI) data showed that a 3% increase in myocardial fibrosis area was associated with a 1.81 (95% confidence interval 1.53–2.13)-fold increase in the risk of mortality [3].

According to Yamada, T. et al. the degree and type of fibrosis can be considered as a predictor of the effectiveness of long-term therapy for chronic heart failure with β-adrenoblockers [4].

In view of the above, clinical experts often suggest that the degree of myocardial fibrosis should be identified and assessed. This will be useful not only in terms of understanding the clinical picture of most cardiovascular diseases but also, in some cases, for the effective prognosis and adequate treatment of patients. However, at present there are no generally accepted classifications of the severity of fibrosis and their clinical correlates, which complicates the objective assessment of this condition.

Endomyocardial biopsy (EMB) is the gold standard for diagnosing many myocardial diseases, including primary cardiomyopathies, myocarditis, and drug or autoimmune myocardial lesions. EMB is pivotal in managing patients with unexplained acute HF accompanied by hemodynamic instability or ventricular arrhythmias/conduction disturbances of unknown etiology [5]. It is also crucial for those with clinically suspected autoimmune myocarditis, cardiac sarcoidosis, storage diseases, infiltrative diseases, cardiac tumors, and in monitoring rejection status in heart transplant patients. Endomyocardial biopsy is an accurate diagnostic tool for restrictive cardiodesminopathies, myocardial iron overload (both intramyocellular as seen in HFE hemochromatosis and mitochondrial in Friedreich’s ataxic cardiomyopathy), cystinosis, and lysosomal storage diseases such as Fabry disease [6]. When interpreting GLA mutations or initiating therapy is contentious, an EMB may be necessary, thus providing definitive evidence of AFD by demonstrating fine granular vacuolization via Sudan black staining, concentric lamellar bodies formed by Gb3, and typical lysosomal inclusions or “zebra” bodies under electron microscopy. The vacuolization and presence of lamellar bodies identified through light and electron microscopy, respectively, are considered histological hallmark findings [7]. Beyond its diagnostic value, virus detection through immunohistochemistry, PCR, reverse transcription PCR (RT-PCR), and the direct sequencing of heart samples have therapeutic implications, such as evaluating potential candidates for immunosuppressive therapy. Moreover, traditionally, a EMB has been the only method to visualize and measure the collagen volume fraction in the myocardium [8,9]. The aim of the study was to determine the significance of ventricular endomyocardial biopsies for the assessment of myocardial fibrosis and to identify the severity of myocardial fibrosis in different cardiovascular diseases.

## 2. Methods

A retrospective study with histologic examination of the results of endomyocardial biopsies of 20 patients with chronic lymphocytic myocarditis (CM), 21 patients with hypertrophic cardiomyopathy (HCM), and 36 patients with a long history of hypertensive and ischemic heart disease (HHD + IHD) was performed. The results were compared with control group data from EMBs taken on 12–14 days after heart transplantation (n = 28). Also, for 1 patient without clinical and morphologic data for cardiovascular pathology, myocardial fragments were taken post mortem from typical sites of EMB and septal reduction sites. EMBs from the right ventricle and interventricular septum, as well as endomyocardial tissue after septal reduction, were included in the study. Each of the biopsy specimens was evaluated separately. A total of 2 to 6 biopsy specimens were obtained per EMB sampling procedure. All EMBs studied were divided into three groups according to the clinical and morphologic diagnosis.

Group I (number of EMBs n = 92, number of patients n = 20) patients with chronic lymphocytic myocarditis (CM) confirmed by immunohistochemical examination according to the position of the European Society of Cardiology Working Group on Myocardial and Pericardial Diseases [10]. Among the patients of group I, there were 8 patients in whom EMBs were taken repeatedly in order to evaluate the treatment dynamics (number of EMBs n = 66) with the time interval between sampling from 89 to 531 days.

Group II (number of patients n = 36, number of endomyocardial fragments obtained during septal reduction n = 65) patients with a long history of CVD, including hypertension (HHD) and ischemic heart disease (IHD). Echocardiographic findings in all patients revealed left ventricular systolodiastolic dysfunction with left ventricular outflow tract subobstruction in the absence of HCM criteria and glycogenosis. Morphologic study of myocardial specimens after septal reduction did not provide histologic evidence for HCM.

Group III (number of patients n = 12, number of endomyocardial fragments obtained during septal reduction n = 21) patients with echocardiographic evidence of obstructive hypertrophic cardiomyopathy (HCM) confirmed by morphologic criteria according to myocardial examination after septal reduction [11].

Also in the study, the control group was created from EMBs of the right ventricle and interventricular septum (number of patients n = 28, number of EMBs n = 61) taken 12–14 days after heart transplantation in order to assess the crisis of cellular and humoral rejection of the graft. Only EMBs without histologic signs of cellular and humoral rejection were included in the control group.

All EMB samples included in the study were stained with either van Gieson’s picrofuchsin or Masson’s trichrome using reagents from BioVitrum (Saint Petersburg, Russia). The area of fibrosis was calculated by one specialist on scanned histological preparations using Aperio AT 2 histoscanner (Vista, CA, USA). For calculating the relative fibrosis area, we manually outlined the connective tissue fields in the myocardium using Aperio ImageScope software 12.1 (Leica, Vista, CA, USA) and summed the resulting area values. The relative fibrosis area was then calculated as the ratio of the total area of collagen fibers to the total biopsy area, excluding the endocardium and subendocardial fibrosis if present, and was expressed as a percentage.

In addition, in 1 patient without clinical and morphologic evidence of CVD who died of ischemic cerebral infarction with subsequent edema and dislocation, post mortem myocardial fragments were taken from the right ventricular apex, middle third of the free wall of the right ventricle, lower third of the interventricular septum on the right ventricular side, lower third of the interventricular septum on the left ventricular side, from the interventricular septum of the left ventricular outflow tract, and from the free wall of the left ventricular outflow tract. The obtained histological preparations were also stained with Masson trichrome; the relative area of fibrosis was calculated.

Statistical analysis was performed in Jupyter Notebook in Python 3.9 programming language using Pandas, Matplotlib, Scipy, Scikit_posthocs, Sklearn, and Numpy libraries. The normality of the distribution was assessed using the Shapiro–Wilk test. All distributions studied were different from normal. Wilcoxon’s test was used to compare dependent samples. Kruskal–Wallis H test and Dunn’s test with Bonferroni-corrected *p* values were used for multiple comparisons of independent samples; ROC analysis was used to determine the success of binary classification. Differences were considered statistically significant at *p* < 0.05.

## 3. Results

The relative area of fibrosis in EMBs in the group of patients with CM was 5.6 [3.3; 12.6]%. At the same time, biopsy specimens with an almost complete absence of collagen fibers (less than 1.0% of biopsy area) and foci of substitutive cardiosclerosis were observed in 14 (15%) cases. In the group of patients with CVD, the relative area of fibrosis was 11.1 [6.6; 15.9]%. However, no biopsy specimens with an almost complete absence of fibrotic tissue were recorded in any case; foci of substitution cardiosclerosis were documented in seven (11%) biopsy specimens. In the HCM patient group, the relative area of fibrosis was 13.4 [8.8; 16.7]%. Single biopsy specimens with minimal amounts of fibrotic tissue were also observed. Foci of replacement cardiosclerosis were noted in 3 (14%) EMBs. The relative area of fibrosis in the control group of patients was 2.7 [1.5; 4.6]%. In the majority of cases in the EMBs, fibrosis was minimal (less than 1%), while one case had a focus of substitution cardiosclerosis (Table 1).

**Figure 1:**
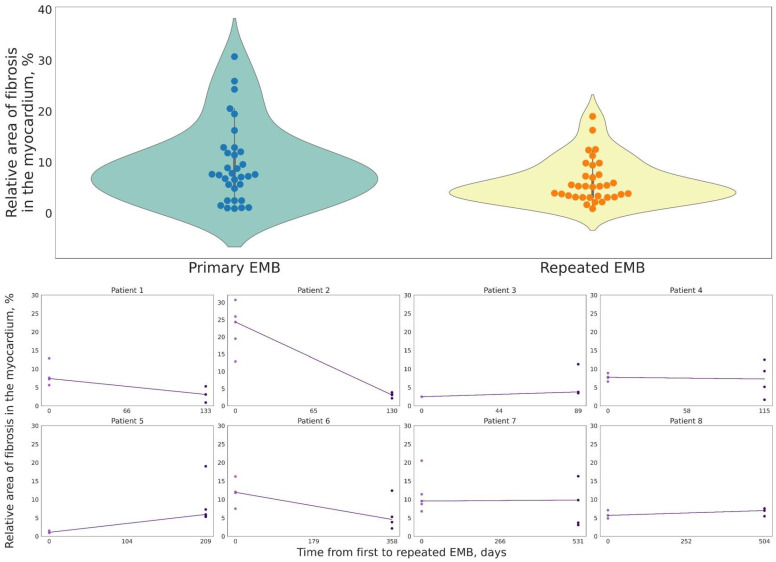
Relative area of fibrosis the myocardium in patients with chronic myocarditis, observation in dynamics (repeated EMBs of 8 patients).

**Figure 2:**
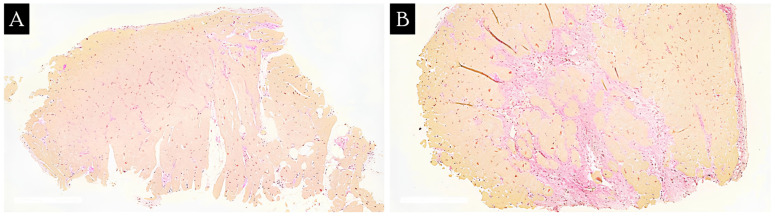
Representative histological slides of two biopsy specimens from the same patient with different relative areas of fibrosis using van Gieson’s picrofuchsin staining at ×100 magnification. (**A**)—relative area of fibrosis: 3.3%. (**B**)—relative area of fibrosis: 18.9%.

When comparing the relative fibrosis area of CM patients in repeat EMBs, it was found to be 7.6 [4.8; 12.0]% in the first EMBs and 5.3 [3.2; 7.6]% in repeat EMBs. However, no statistically significant differences in the fibrosis area were found between primary and recurrent EMBs (*p* = 0.15). There was no increased incidence of replacement cardiosclerosis in repeat EMBs compared to primary EMBs. In addition, in four of eight cases, we observed less severe fibrosis in repeat EMBs compared to primary EMBs (Figure 1). It should also be noted that in different biopsy specimens from the same patient, the value of the relative area of fibrosis in the EMBs could be either homogeneous with variations of 1–5% or pronounced with variations of 10–20% (Figure 2). The main clinical characteristics of patients whom EMBs were taken repeatedly are shown in Table 2.

**Figure 3:**
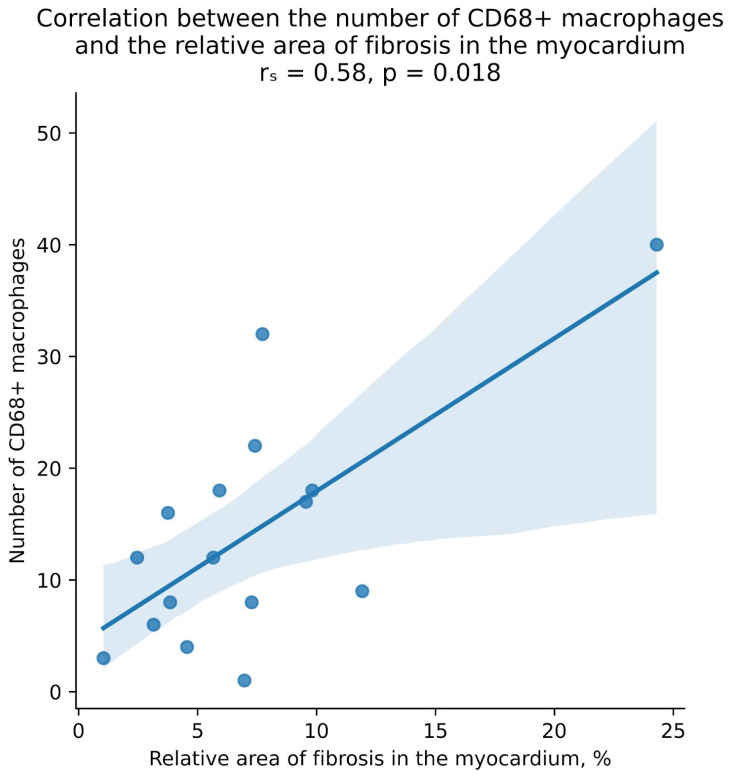
Positive correlation between the number of macrophages in the myocardial interstitium and the severity of fibrosis.

When conducting a correlation analysis in patients with chronic lymphocytic myocarditis, in whom EMBs were taken twice, a positive correlation was found between the level of macrophages in the myocardial interstitium and the relative area of fibrosis (Figure 3).

**Figure 4:**
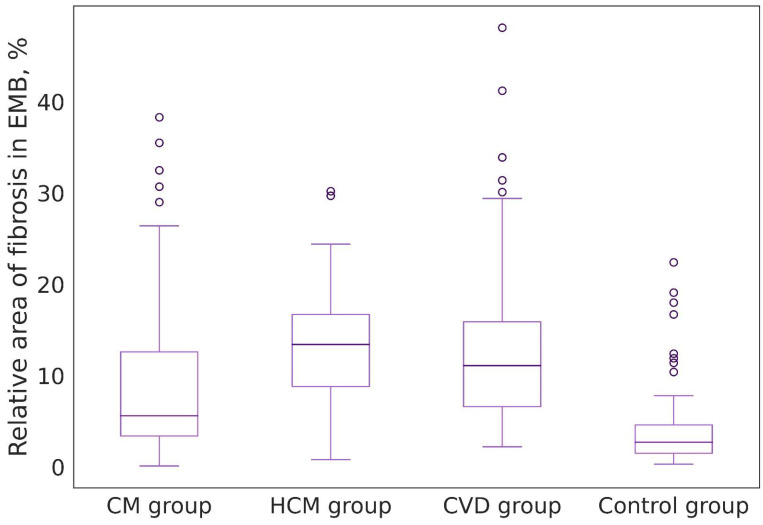
Distributions of the relative area of fibrosis in different groups of patients. CM—chronic myocarditis. HCM—hypertrophic cardiomyopathy. CVD—cardiovascular diseases (Hypertensive Heart Disease and Ischemic Heart Disease).

When all patients were compared, statistically significant differences were found between groups (*p* = 1.3 × 10^−13^). We observed more pronounced differences between patients in the control group and the remaining groups, where the median fibrosis area was 2.9% lower compared to patients with CM; 8.4% lower compared to patients with CVD; and 10.7% lower compared to patients with HCM. Differences were also found between patients with CM and patients with HCM, with the latter having a median fibrosis area that was 7.8% higher; and patients with CVD had a median area 5.5% higher compared to patients with CM. No differences were found between patients in the HCM group and patients with CVD (Figure 4).

**Figure 5:**
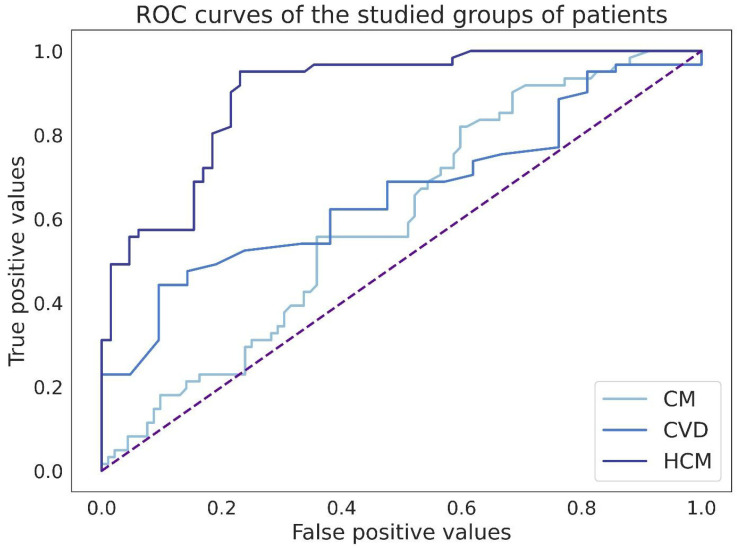
ROC curves of the studied groups of patients. CM—chronic myocarditis. HCM—hypertrophic cardiomyopathy. CVD—cardiovascular diseases (Hypertensive Heart Disease and Ischemic Heart Disease).

When the ROC curves were plotted to determine the success of the binary classification between the control and study groups, it was found that a relative area of fibrosis in the myocardium of 1.1% or less was highly specific for patients whose EMBs were harvested 12–14 days after heart transplantation compared to the study groups. Thus, compared to CM patients, the specificity of this fibrosis area value was 95.7%; compared to CVD patients, it was 95.3%; and compared to HCM patients, it was 100% (Figure 5).

**Figure 6:**
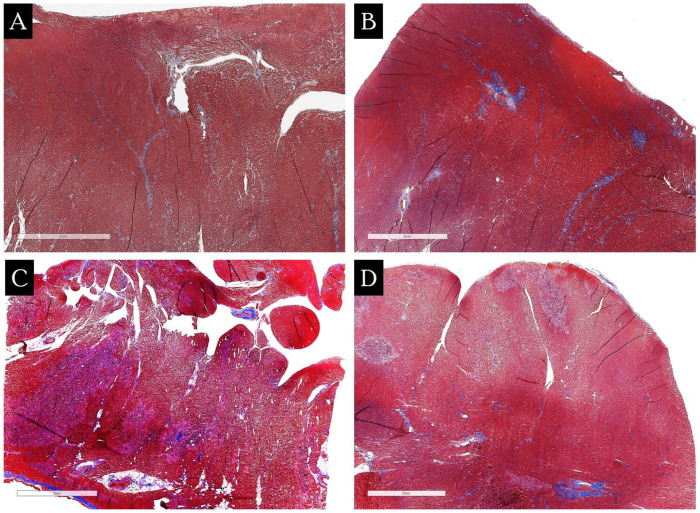
Overview histological slides of the myocardium, Masson’s trichrome staining, ×10. (**A**)—myocardium of the lower third of the interventricular septum from the right ventricle (0.88%); (**B**)—myocardium of the middle third of the right ventricle (1.30%); (**C**)—myocardium of the free wall of the left ventricle in the area of the outflow tract (2.79%); (**D**)—myocardium of the interventricular septum in the area of the outflow tract (2.01%). The relative area of fibrosis is indicated in parentheses.

In the study of myocardial fragments taken at autopsy from a patient without CVD, the relative area of fibrosis at the typical EMB site was as follows: in the lower third of the right ventricular free wall, it was 1.3%; in the lower third of the interventricular septum on the right ventricular side, it was 0.9%. The relative area of fibrosis at the typical site of septal reduction in the outflow tract of the interventricular septum was 2.01%, and in the area of the left ventricular free wall of the outflow tract, it was 2.8%. In the lower third of the interventricular septum on the left ventricular side, the percentage of fibrous tissue in the myocardium was 1.9%, and in the right ventricular apex, it was 2.5% (Figure 6).

**Figure 7:**
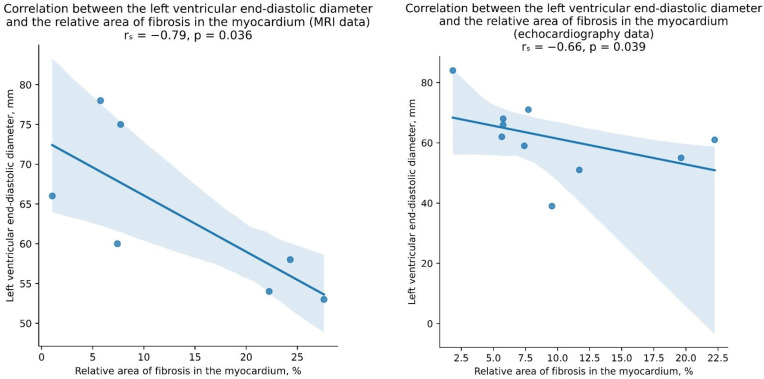
Negative correlations between LVEDD calculated from MRI and echocardiography and the relative area of fibrosis in the EMB.

We additionally analyzed MRI data for 8 patients in Group I and echocardiography data for 10 patients in Group I. For the MRI analysis, the following parameters were evaluated: LV end-diastolic diameter (LVEDD), LV end-systolic diameter (LVESD), LV end-diastolic volume (LVEDV), LV end-systolic volume (LVESV), ejection fraction (EF), left ventricular stroke volume (LVSV), cardiac output, right ventricular wall thickness, right ventricular ejection fraction, and the mention of myocardial fibrosis in the MRI report. A statistically significant negative correlation was found only between the LVEDD and the relative area of fibrosis in the EMBs (r_s_ = −0.79, *p* = 0.036). It is also worth noting that in all cases examined, the presence of fibrosis was mentioned in the MRI report. For the echocardiographic analysis, the following parameters were considered: the myocardial mass index, LVEDD, LVESD, LVEDV, LVESV, EF, and TAPSE. Similar to the MRI data analysis, a statistically significant negative correlation was found only between the LVEDD and the relative area of fibrosis in the EMBs (r_s_ = −0.66, *p* = 0.039) (Figure 7).

However, these correlations should be interpreted with caution due to the small sample size and variability in the assessment of fibrosis based on the EMB material.

## 4. Discussion

Our study found that in different EMBs from the same patient, the severity of myocardial fibrosis could differ significantly in all studied patient groups. Moreover, at present, there are still no validated scales for myocardial fibrosis assessment, and many authors assess and interpret the severity of fibrosis using their own conventional scales. The volume of connective tissue in the norm is also controversial.

According to the study by Oken D.E. et al., in which fibrotic tissue was determined using the biochemical method by the estimation of hydroxyproline concentration, the collagen content in the chambers of a normal heart weighing 250 g in the left ventricle was 1.15 g, in the right ventricle was 0.83 g, in the left atrium was 0.45 g, and in the right atrium was 0.67 g. Thus, the average percentage of connective tissue in the myocardium in this study was determined as 1.2% [12].

In the study by Mendes A.B. et al., the percentage of fibrous tissue in the myocardium of the anterior wall of the left ventricle of patients of different ages was compared, and it was found that in children the collagen content was 2.1 ± 0.1%; in adults was 3.2 ± 0.2%; and in the elderly was 5.6 ± 0.3% [13].

In the Miles C. et al. study, an automatic calculation of the collagen percentage was performed using artificial intelligence software (Visiopharm A/S, Hoersholm, Denmark). In this study, the percentage of connective tissue was calculated not only in the myocardium but also in the epicardium and endocardium. Thus, according to the results of the study, the fibrous tissue content was 15.2 (13.2–17.5)% in the myocardium of the right ventricle, 8.6 (7.4–9.9)% in the interventricular septum, and 9.5 (8.2–10.9)% in the right ventricle [14].

The results of our study partially agree with the two previous studies: the relative connective tissue area of the right ventricular myocardium and interventricular septum of cardiac donors without clinically significant concomitant CVD was 1.1%, with a specificity of more than 95% compared to patients with the presence of CVD. However, we cannot exclude the fact that donors had a subclinical course of CVD or were inadequately screened, which is a limitation to the interpretation of the findings. Differences in the percentage of fibrotic tissue in the myocardium in patients with CM and in patients with HCM and CVD can be partially explained by different anatomic localizations of the material examined.

Thus, in the points of typical EMB sampling, the detected percentage of fibrous tissue correlates with that detected in ROC analysis of all groups of patients (1.1%), while in the region of the right ventricular apex, we saw a sharp increase in the content of dense fibrous tissue in the myocardium, which can be explained by anatomical features. In the region of the left ventricular outflow tract, we saw that the relative area of fibrosis was almost two times greater, which should also be extrapolated to the concept of “normality” of the content of dense fibrous connective tissue in this anatomical zone. Thus, a relative fibrotic tissue area of 3% or less is highly specific for normal myocardium compared to patients with HCM, where the specificity was 95.24%, and to patients with CVD (specificity 96.92%). However, in our opinion, writing everything off to the peculiarities of connective tissue distribution in normal myocardium would be wrong, given the data of previous investigators. The lower amount of fibrous tissue in patients with CM, as well as “reduction” of fibrous tissue in repeated EMBs in such patients, may be due to the focal involvement of myocardial tissue in the process. From the clinical point of view, the obtained data may indirectly indicate a less pronounced contribution of fibrosis to the pathogenesis of HF in patients with CM, where active inflammation, necrosis, and the dystrophy of cardiomyocytes come to the fore. The influence of the biopsy sample size on the final percentage of cardiomyocyte replacement by fibrous tissue cannot be excluded: the study by Meckel C.R. et al. showed that the area of myocardial fibrosis became reliably smaller as the size of the tissue sample increased [15].

According to the results of the study of the heart without signs of CVD, we found that the relative area of fibrosis in the region of the right ventricular apex was almost two times higher than in its free wall and in the interventricular septum on the right ventricular side. The obtained data may also explain the variability in the distribution of fibrosis in the EMBs, especially in the control group.

In contrast to CM, the key link in the pathogenesis of HF in patients with HCM and CVD seems to be the progressive accumulation of collagen in the myocardial interstitium, which leads to the formation of systolodiastolic dysfunction [16]. This postulate has been confirmed in other studies, where it was shown that myocardial collagen volume content was increased in patients with HF compared to controls by 7.51 ± 0.49% and 1.95 ± 0.07%, respectively [17].

It should also be emphasized that in most cases, fibrosis in patients with CVD and HCM is localized subendocardially and is rarely transmural. This can be explained by the presence of a transmural pressure gradient and the reorganization of myocardial microcirculation, especially in HCM, thus contributing to chronic ischemia of the subendocardial zones. In addition, hypoxia in the case of marked myocardial hypertrophy is also caused by a mismatch of the coronary artery perimeter with the heart weight.

In addition to the relative area of fibrosis, the collagen composition of fibers and their physicochemical properties are also important for the pathogenesis of heart failure in CVD. It was shown that type I collagen prevailed in the myocardium of patients with clinical HF on the background of hypertension, and in HF on the background of HCM, the ratio of type I and III collagen was approximately equal [18,19].

There is still no consensus on the interpretation of myocardial fibrotic tissue area data. For example, in the study of AokiT. et al., the level of fibrosis in the myocardium of 0.48% was interpreted as moderate, and 3.3% was interpreted as severe [20]. In another study, myocardial connective tissue area of 3.3 [2.6–6.1]% was considered normal in a control group of patients without macroscopic signs of CVD, while fibrosis in 16% was evaluated as moderate, and fibrosis in 70% was evaluated as severe [21]. Also, one study showed a strong association between the relative area of myocardial fibrosis and the presence of atrial fibrillation; in particular, it was shown that the percentage of collagen fibers was more than 2-fold higher in patients with atrial fibrillation than in patients without fibrillation [22].

The obtained data may indicate that persistent rhythm and conduction disorders lead to the predominance of collagen synthesis over its degradation in myocardium, which we can clinically see in the form of the development of so-called “arrhythmia-induced cardiomyopathies”.

Thus, disagreements in the literature regarding the interpretation of data on the “normal” ratio of collagen fibers to cardiomyocytes, the absence of validated scales for assessing the severity of fibrosis, ambiguity in the interpretation of the values obtained, variability in the severity of fibrosis in EMBs even within one patient, and the dependence on the localization of material collection make the morphological study of EMBs unpromising as a universal and widely reproducible assessment method. Moreover, EMB with regard to the degree of fibrosis is practically impossible to use for the purposes of dynamic monitoring and control of therapy, both due to the above-described limitations and due to the invasiveness of the procedure and the need for preliminary preparation of the patient.

The view of the modern clinician should be directed to the search for more universal and less invasive methods of cardiac fibrosis assessment. Thus, cardiac MRI should be considered as an alternative to EMB, with examination of myocardial interstitium being performed by measuring its extracellular volume using T1 mapping. According to a metaanalysis of 15 large correlation studies, an overall positive high correlation of r = 0.88 (95% confidence interval: 0.854–0.914) was found between EMB and MRI data [23]. In another study, no correlations were found between myocardial collagen volume fraction and T1-mapped weighted myocardial images, but a weak to moderate correlation was found between T1-mapped extracellular volume and left ventricular ejection fraction, the global longitudinal strain, and the NTproBNP [21]. This result could be due to the heterogeneity of myocardial fibrosis, which shows marked variability of fibrosis area in different EMBs from the same patient and a specificity of material sampling during surgery. At the same time, MRI data clearly showed a correlation with functionally and prognostically more significant indices, which also confirms the possibility of more widespread use of MRI studies to detect indirect signs of myocardial fibrosis.

In the study of Kasner M. et al., it was shown that the time of early diastolic filling deceleration measured during tissue Dopplerography had a weak correlation r = 0.43 with the area of fibrous tissue measured from myocardial EMB in patients with heart failure with preserved ejection fraction [24]. However, for wide application of the above techniques in practice, large-scale basic research is required. In particular, it is possible to perform studies using 3D morphometry of the myocardium in comparison with MRI and echocardiography images [25]. The collagen concentration can also be measured by biochemical methods. The analysis of hydroxyproline and a collagen-specific amino acids provides a reliable estimate of the collagen concentration. Soluble and insoluble fractions of the total collagen pool can be identified using this approach. However, this simple analysis of collagen concentration does not determine the topography of the collagen fiber arrangement and, as a consequence, does not allow us to draw conclusions regarding possible pathogenetic mechanisms of excessive fibrillogenesis [26].

The question of the reversibility of fibrotic changes in myocardium remains not fully understood. It is widely known that fibrosis can be conditionally divided into two types: substitutive (reparative) and diffuse (remodeling) fibrosis. The former develops due to direct replacement of necrotized tissues, while the latter is etiologically related to a large spectrum of causes, including chronic hypoxia, autoimmune damage, intoxication, pressure and/or volume overload of the heart chamber, and some others. This type of fibrosis is characterized by the diffuse increase of fibrous tissue in the myocardial interstitium and excessive collagen deposition at the periphery of the intramyocardial vessels. Many authors agree that the development of replacement cardiosclerosis is currently irreversible, and the use of stem cells is currently limited by their low ability to differentiate into cardiomyocytes compared to differentiation into fibroblasts [27,28].

Interstitial diffuse fibrosis is thought to affect the consistency between myocardial excitation and contraction during both systole and diastole events. Increased interstitial collagen deposition in the perimysium is also associated with increased ventricular stiffness and the development of diastolic dysfunction. Moreover, active fibrotic remodeling of the interstitium leads to the formation of capillary–parenchymal blockage, chronic hypoxia of the cardiomyocytes, and a decrease in their functional activity. Also, excessive collagen accumulation in the interstitium can disrupt the coordinated contraction of muscle fiber bundles, as well as provoke sliding displacement of the cardiomyocytes, which leads to a functional reduction in the number of muscle layers in the ventricular wall and to the subsequent dilatation of the left ventricle [29]. It is also important to emphasize that in the development of replacement fibrosis, an important role is played by profibroblasts migrating from peripheral blood and differentiating in tissues to mature fibroblasts, while in remodeling cardiosclerosis, the leading role in fibrillogenesis is attributed to resident myocardial fibroblasts and myofibroblasts. It is believed that it is resident fibroblasts that can become a potential therapeutic target in an attempt to slow down the progression of cardiac fibrosis or, in the boldest studies, even contribute to reverse remodeling of the myocardial interstitium [29,30].

The role of monocytic macrophage cells and T-lymphocytes in the possible activation of resident myocardial fibroblasts with subsequent excessive fibrillogenesis is also being actively studied [31]. This line of research also has great potential significance in the context of developing therapies aimed at preventing the development of fibrosis in the myocardium.

Recently, there has been much evidence that novel coronavirus infection may damage not only the respiratory tract but also the cardiovascular system. Many authors have pointed to vascular-mediated myocardial damage in the acute period of infection with the development of hemorrhage, necrosis, and thrombosis, which will inevitably lead to the formation of foci of replacement fibrosis [32,33,34,35,36,37]. Moreover, in the postacute period of coronavirus infection in a part of patients, we can also see persisting damage in the myocardium, which can potentially lay the foundation for the formation of autoinflammatory diseases and promote progressive collagen deposition in the interstitium [38]. Also, impaired T-cell function has been observed in the background of coronavirus infection, thus potentially leading to overstimulation of myocardial myofibroblasts and contributing to the development of a profibrotic state [39,40,41,42,43]. Given that we are still only collecting data on the cardiovascular effects of SARS-CoV-2, additional time is required to fully evaluate the progression of cardiovascular disease on the COVID-19 background and to clarify the role of infection in the development of myocardial fibrosis [43].

## 5. Conclusions and Future Perspectives

EMB in the assessment of myocardial fibrosis has a questionable role due to the heterogeneity of fibrotic changes in the myocardium. To adequately assess the severity of fibrosis in various CVDs, it is necessary to perform large-scale clinical and morphologic studies with stereotypical sampling of the myocardial tissue fragments in comparison with echocardiography and MRI data, as well as to perform 3D morphometry of the myocardium.

## 6. Study Limitation

Our study did not assess collagen fiber crosslinking density, use biochemical detection methods for this collagen type, or include endpoint observations for patients. Thus, we cannot draw conclusions regarding the dynamics of the development of fibrosis in the myocardium and its prognostic significance for a patient. Echocardiographic data on the right ventricular free wall longitudinal strain, left ventricular global longitudinal strain, and E/e’ ratio were not available and were not included in the study.

## Figures and Tables

**Table 1:** Relative area of fibrosis in the EMB in different groups of patients.

Groups of Patients	Value Range (%)	Median, 25%, and 75% Percentiles (%)	Foci of Replacement Cardiosclerosis in Biopsy Specimens (n/%)
Group I: EMBs of patients with chronic lymphocytic myocarditis (n = 20)	0.1–38.3	5.6 [3.3; 12.6]	14 (15.2)
Group II: Endomyocardial fragments obtained during septal reduction of patients with a long history of coronary heart disease combined with hypertension (n = 36)	2.2–48.1	11.1 [6.6; 15.9]	7 (10.7)
Group III: Endomyocardial fragments obtained during septal reduction of patients with hypertrophic cardiomyopathy (n = 12)	0.8–30.2	13.4 [8.8; 16.7]	3 (14.3)
Control group: EMB of patients on 12–14 days after heart transplantation (n = 28)	0.3–22.4	2.7 [1.5; 4.6]	1 (1.6)

**Table 2:** Clinical characteristics of patients of group I for whom EMBs were taken repeatedly (n = 8).

Patient	Age	Gender	Time between EMB, Days	Dynamics of LVEF, %	Dynamics of TAPSE, in mm	Dynamics of CD3+ Cells in the Inflammatory Infiltrate	Dynamics of CD68+ Cells in the Inflammatory Infiltrate	Dynamics of VP1-EntV Expression in Cardiomyocytes, %	Dynamics of the Relative Area of Fibrosis in the Myocardium, %	Specific Therapy	Treatment of HF and Concomitant Pathology
1	28	F	133	49 vs. 61	16 vs. 20	46 vs. 15	22 vs. 8	80 vs. 0	7.41 vs. 3.45	Prednisolone: starting dose 30 mg with dose reduction to 15 mg Human immunoglobulin normal 0.4 g/kg	β-adrenoblocker ACE inhibitor Rivaroxaban Torasemide Spironolactone
2	32	M	130	38 vs. 48	17 vs. 19	19 vs. 14	40 vs. 6	80 vs. 20	24.30 vs. 3.15	Prednisolone: starting dose 30 mg with dose reduction to 15 mg	β-adrenoblocker Digoxin Rivaroxaban Amiodarone Torsemide Molsidomine
3	31	F	89	42 vs. 51	14 vs. 15	18 vs. 8	12 vs. 16	0 vs. 0	2.46 vs. 3.76	Prednisolone: starting dose 30 mg with dose reduction to 15 mg	β-adrenoblocker ACE inhibitor Spironolactone
4	26	F	115	12 vs. 15	13 vs. 17	18 vs. 3	32 vs. 8	0 vs. 0	7.73 vs. 7.27	Prednisolone: starting dose 1 mg/kg with dose reduction to 15 mg Mycophenolate mofetil 2 g/day	β-adrenoblocker Valsartan + Sacubitril Apixaban Amiodarone Torsemide Spironolactone
5	41	M	209	19 vs. 33	13 vs. 17	26 vs. 4	3 vs. 18	90 vs. 20	1.05 vs. 5.92	Prednisolone: starting dose 30 mg with dose reduction to 15 mg	β-adrenoblocker ACE inhibitor Spironolactone Warfarin Levosimendan three times
6	33	M	358	58 vs. 64	14 vs. 15	25 vs. 5	9 vs. 4	100 vs. 50	11.92 vs. 4.56	None	β-adrenoblocker ACE inhibitor Acetylsalicylic acid
7	59	F	531	16 vs. 18	11 vs. 17	50 vs. 20	17 vs. 18	80 vs. 50	9.56 vs. 9.82	Prednisolone: starting dose 30 mg with dose reduction to 15 mg Human immunoglobulin normal 0.4 g/kg	β-adrenoblocker ACE inhibitor Spironolactone Rivaroxaban Amiodarone Torsemide
8	30	M	504	60 vs. 48	16 vs. 17	15 vs. 1	12 vs. 1	0 vs. 0	5.66 vs. 6.97	None	β-adrenoblocker ACE inhibitor

## Data Availability

All source data used in this study are available from the corresponding author upon request; if you need clarifications or need additional information, you can write to the following email: doctormakarovia@gmail.com.

## Abbreviations

CM	chronic myocarditis
CVD	cardiovascular disease
EMB	endomyocardial biopsy
HCM	hypertrophic cardiomyopathy
HF	heart failure
HHD	hypertensive heart disease
IHD	ischemic heart disease
MRI	magnetic resonance imaging
